# Identification of Three Antiviral Inhibitors against Japanese Encephalitis Virus from Library of Pharmacologically Active Compounds 1280

**DOI:** 10.1371/journal.pone.0078425

**Published:** 2013-11-04

**Authors:** Jin'e Fang, Leqiang Sun, Guiqing Peng, Jia Xu, Rui Zhou, Shengbo Cao, Huanchun Chen, Yunfeng Song

**Affiliations:** 1 State Key Laboratory of Agricultural Microbiology, Huazhong Agricultural University, Wuhan, China; 2 College of Veterinary Medicine, Huazhong Agricultural University, Wuhan, China; Utah State University, United States of America

## Abstract

Japanese encephalitis virus (JEV) can cause severe central nervous disease with a high mortality rate. There is no antiviral drug available for JEV-specific treatment. In this study, a cytopathic-effect-based, high-throughput screening assay was developed and applied to screen JEV inhibitors from Library of Pharmacologically Active Compounds 1280. The antiviral effects of three hit compounds including FGIN-1-27, cilnidipine, and niclosamide were evaluated in cells by western blotting, indirect immunofluorescence assay, and plaque reduction assay. A time-of-addition assay proved that all three compounds inhibited JEV at the stage of replication. The EC50s of FGIN-1-27, cilnidipine, and niclosamide were 3.21, 6.52, and 5.80 µM, respectively, while the selectivity indexes were 38.79, 30.67, and 7.49. FGIN-1-27 and cilnidipine have high efficiency and selectivity against JEV. This study provided two JEV antiviral inhibitors as candidates for treatment of JEV infection.

## Introduction

Japanese encephalitis virus (JEV), a member of the genus *Flavivirus* in the family *Flaviviridae*, is a mosquito-transmitted and zoonotic pathogen that causes 50,000 cases and 10,000 deaths per year [1]. There are >70 arboviruses in the genus *Flavivirus* including JEV, dengue virus (DENV), West Nile virus (WNV), and yellow fever virus [2]. JEV can cause severe central nervous disorders such as poliomyelitis-like paralysis, aseptic meningitis, and encephalitis in humans. The fatality rate caused by JEV is 10–50% and half of the survivors have severe neurological sequelae, including persistent motor defects and severe cognitive and language impairments [3]. The geographic range of JEV is still expanding with an enhanced threat, and JEV infections have been reported in Australia [4], [5], Pakistan [6], and Saipan [7] in the past 30 years. Therefore, JEV is still an important pathogen that has global health significance.

Inactivated and live-attenuated vaccines have been used for prevention of JEV infection for many years [8], [9]. Although vaccines have reduced the incidence of JE in some countries, they seem not to be effective against all the clinical isolates [10]. In August 2006, there was an outbreak of JEV in Shanxi Province, China, which caused 66 cases and 19 deaths [11]. There is an urgent need for antiviral agents that can reduce the death toll and neurological sequelae of JEV infection [12]. Two anti-hepatitis C virus drugs targeting viral protease, telaprevir VX-950 (developed by Vertex) and boceprevir SCH503034 (developed by Merck), won approval in 2011 [13]. Various effective inhibitors against DENV and WNV have also been identified as drug candidates [14], [15]. In recent studies, some agents were found to have good antiviral effects against JEV. Indirubin, derived from *Isatis indigotica* extract, was proved to have inhibitory effects on JEV in *vitro* with less cytotoxicity [16]. Dehydroepiandrosterone (DHEA) suppressed the replication and virus-induced apoptosis in neuroblastoma cells by acting on the extracellular signal-regulated protein kinase [17]. N-nonyl-deoxynojirimycin affected the interaction between calnexin (endoplasmic reticulum chaperone) and JEV glycoproteins (premembrane, envelope, and non-structural protein 1), and thus had anti-JEV effects both *in vitro* and *in vivo* [18]. SCH 16, a derivative of N-methylisatin-β-thiosemicarbazone, inhibited 50% of the plaques produced by JEV at a concentration of 16 µg/mL (0.000025 µM) [19]. However, there are currently only a small number of JEV inhibitors available for drug development.

In this study, a cytopathic-effect-(CPE)-based, high-throughput screening (HTS) assay was developed for discovery of JEV antiviral inhibitors. It was used to screen 1280 pharmacologically active compounds and three compounds were identified to have antiviral effects against JEV.

## Materials and Methods

### Cell and virus

BHK-21 cells were cultured in Dulbecco's Modified Eagle's Medium (Sigma-Aldrich, St. Louis, MO, USA) supplemented with 10% fetal calf serum (FCS) (Invitrogen, Grand Island, NY, USA), 100 U/mL penicillin (Sigma-Aldrich), and 100 µg/mL streptomycin (Sigma-Aldrich). JEV (P3 strain, Genbank accession no. U47032.1) was propagated in BHK-21 cells with maintenance medium containing 1% FCS, 100 U/mL penicillin, and 100 µg/mL streptomycin.

### Cell viability assay

Cell viability was evaluated by Celltiter-Glo Luminescent Cell Viability Assay reagent (Promega, Madison, WI, USA) following the manufacturer's protocol. An equal volume of Celltiter-Glo reagents was added to the cells in 96-well white plates (Corning, Tewksbury, MA, USA) and mixed for 2 min on an orbital shaker and incubated for a further 10 min at room temperature. The luminescence of each well was measured by a 1450 MicroBeta TriLux (Perkin Elmer, Waltham, MA, USA). Percentage of cell viability was calculated as follows: Percentage of cell viability  = 100× (luminescence of experimental group/luminescence of control group).

### Optimization of HTS assay conditions

The cell density, assay endpoint, and infective dose in the HTS assay were optimized. BHK-21 cells at different densities (5,000–25,000 cells per well) were infected with JEV at various multiplicity of infections (MOIs) (0.64–0.0025). Cell viability was detected at different times (72–120 h) after JEV inoculation. The suitable cell density, assay endpoint, and infective dose for HTS assay were selected by comparing cell growth, S/B ratio, and *Z*′ value in different conditions. The *Z*′ value and S/B ratio were calculated as previously described [20].

### HTS of Library of Pharmacologically Active Compounds 1280

BHK-21 cells were seeded onto 96-well plates at 10,000 cells per well. After 12 h incubation, the culture supernatant was replaced with maintenance medium. One microliter of each compound was added to 99 µL maintenance medium in the first well, followed by twofold serial dilutions for two wells. After full mixing in the third well, 50 µL medium was discarded. Then, 50 µL maintenance medium containing 0.02 MOI JEV was added to each well. The plates were subjected to 30 s horizontal shaking to achieve thorough mixing. Three wells of mock-infected cells as well as three wells of JEV-infected cells containing 1% DMSO were set on each plate as controls. After 120 h incubation, the percentage of CPE inhibition was calculated as previously described [21]: Percentage of inhibition  =  (luminescence of experimental group – average luminescence of virus control) / (average luminescence of cell control – average luminescence of virus control) ×100.

### Identification of antiviral effects of three hit compounds

#### Identification of antiviral effects by western blotting

BHK-21 cells in 12-well plates were infected with JEV at MOI 0.01. The compounds were then added to cells at final concentrations of 10 and 20 µM. After incubation for 48 h, expression of JEV envelope (E) protein was detected by western blotting. The cells were collected after trypsin digestion and washed in PBS. The cell suspension was mixed with an equal volume of 2×SDS sample buffer. The proteins were electrophoresed in 12% SDS-PAGE and transferred to a nitrocellulose filter membrane (Millipore, Darmstadt, Germany). Western blotting was performed with JEV E protein-specific monoclonal antibody (Mab; 1∶500, constructed in our laboratory) or GAPDH-specific Mab (1∶1000; Proteintech, Chicago, IL, USA) as the primary antibodies, followed by addition of horseradish-peroxidase-conjugated goat anti-mouse IgG (Boster, Wuhan, China) as the secondary antibody. Finally, the protein blots were detected with SuperSignal West Pico Chemiluminescent Substrate (Thermo Scientific, Rockford, IL, USA) and imaged by a MF-ChemiBIS 3.2 (DNR Bio-Imaging Systems, Mahale Hahamisha, Israel). The expression of E and GAPDH was quantified by gray-value analysis using software ImageJ. The expression of E protein was normalized by the expression of GAPDH and the changes of E expression were calculated by comparing to the non-compound treated group.

#### Identification of antiviral effects by indirect immunofluorescence assay (IFA)

BHK-21 cells were seeded in 12-well plates and inoculated with JEV at MOI 0.01 after 12 h incubation. The compounds were added to cells at concentrations of 5 or 20 µM. After incubation for a further 48 h, virus replication in the cells was evaluated by IFA. The cells were fixed in formaldehyde for 5 min and were incubated with anti-NS5 Mab (1∶500) [22] and then FITC-conjugated goat anti-mouse antibody (Boster) for 30 min respectively. Finally, the cells were stained with DAPI (Sigma-Aldrich) and imaged by a fluorescence microscope (Olympus IX70; Olympus, Tokyo, Japan).

#### Identification of antiviral effects by plaque reduction assay

BHK-21 cells were plated in 96-well plates at a density of 10,000 cells per well and grown for 12 h for cell attachment. The cells were infected with JEV at MOI 0.01 in the presence of different concentrations (20, 15, 10, and 5 µM) of compounds. Each concentration was assayed in triplicate. Forty-eight hours post-infection, the viruses in each group were harvested by freezing/thawing three times and mixed in a tube. Then, 50 µL virus suspension was inoculated into BHK-21 cells in 12-well plates for the plaque assay, as previously described [23].

### Time-of-addition assay

The antiviral mechanism of compounds was evaluated by time-of-addition assay as previously described [24]. BHK-21 cells were seeded in 96-well white plates at 10,000 cells per well, and then infected with JEV at MOI 0.01 after 12 h incubation. The test compounds at10 µM were added to cells at 1 h pre-infection (−1 h), during infection (0 h), and 1 h post-infection (+1 h). The percentage of inhibition of each group was evaluated at 120 h post-infection.

### EC50 assay

BHK-21 cells were seeded in 96-well white plates at 10,000 cells per well. Twelve hours later, the growth medium was replaced with maintenance medium containing 0.01 MOI JEV as well as different concentrations of compounds. Cell incubation was continued for 120 h, and the percentage of inhibition was measured as described above. The EC50 values were calculated by nonlinear regression analysis.

### Cytotoxicity of compounds

BHK-21 cells were seeded in 96-well white plates at 10,000 cells per well. After 12 h incubation for cell attachment, different concentrations of compounds were added to the cells. Cell viability was tested as described above after 120 h incubation. The 50% cytotoxicity concentration (CC50) was calculated by nonlinear regression analysis.

## Results

### Optimization of HTS assay conditions

The HTS assay conditions including seeding cell density, infective dose, and assay endpoint were optimized by comparing the *Z*′ values and S/B ratios under different conditions. Finally, we chose 10,000 cells per well as the optimized cell density, 0.01 MOI as the optimized infective dose, and 120 h post-inoculation as the endpoint of the HTS. Under the optimized conditions, three independent assays were performed to validate the robustness and reproducibility of the HTS assay. The *Z*′ values in the three repeats were 0.92, 0.93, and 0.97, respectively, and the average was 0.94±0.015. The S/B values were 10.82, 9.65, and 10.94, respectively, and the average was 10.47±0.41. The average coefficient variation (CV) in mock-infected and JEV-infected cells was 1.26±0.45% and 5.72±0.23%, respectively. All the results fitted well with the standard parameters of HTS, which demonstrated that the assay was robust and suitable for large-scale compound screening.

### Compound screening

**Figure 1:**
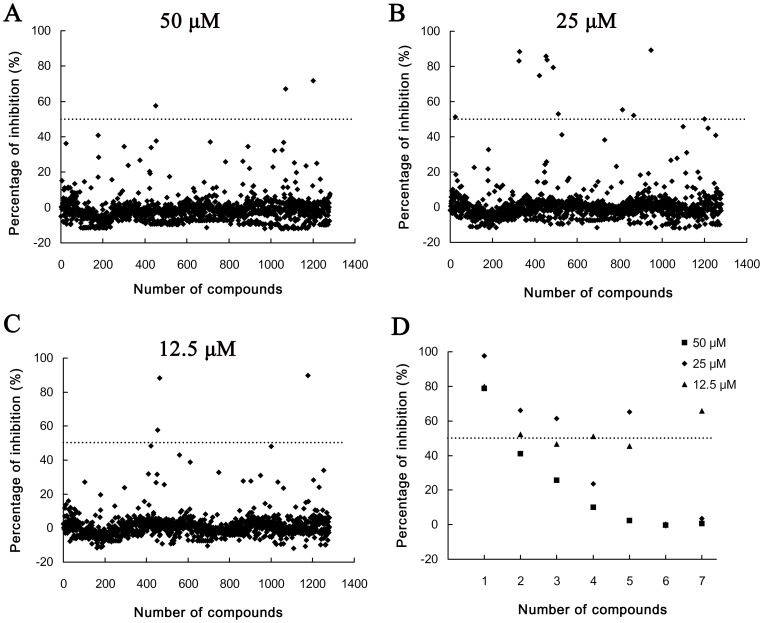
HTS of JEV inhibitors from Library of Pharmacologically Active Compounds 1280. Each dot represents the percentage inhibition of compounds at concentrations of 50 µM (A), 25 µM (B), and 12.5 µM (C). (D) Antiviral effect of seven compounds confirmed by a second screening. Numbers 1–7 in the X axis represent the compounds FGIN-1-27, cilnidipine, niclosamide, UCL 2077, R(+)−butylindazone, palmitoyl-dl-carnitine chloride, and TTNPB, respectively. The dashed lines indicated the 50% inhibition in each panel.

The CPE-based HTS assay was used to screen JEV inhibitors from the Library of Pharmacologically Active Compounds 1280. We obtained an average *Z*′ value of 0.91±0.02, average S/B ratio of 11.9±0.55, and average CV of 1.95±1.76% in mock-infected controls and 7.98±0.99% in JEV-infected cells in this HTS assay. The inhibition rate of each compound at different concentrations was calculated at the end of the assay and plotted in Figure 1A–C. There were 3, 12, and 3 compounds identified to have >50% inhibition at concentrations of 50, 25, and 12.5 µM, respectively. In these compounds, seven compounds were selected for a second screening according three criterions: (1) the compounds had an inhibition rate at about 50%; (2) the compounds showed inhibitory effect to JEV in at least two concentrations; (3) there had normal cells without cytopathic-changes observed by microcopy. After the second screening, four compounds were confirmed to have about 50% CPE inhibition at two concentrations (Figure 1D). The antiviral effects of three commercial available compounds, cilnidipine, FGIN-1-27 (N,N-dihexyl-2-(4-fluorophenyl)indole-3-acetamide), and niclosamide, were identified by western blotting, IFA, plaque reduction assay, and time-of-addition assays in the subsequent studies.

### Antiviral effects of three hit compounds

#### Identification of antiviral effects by western blotting

**Figure 2:**
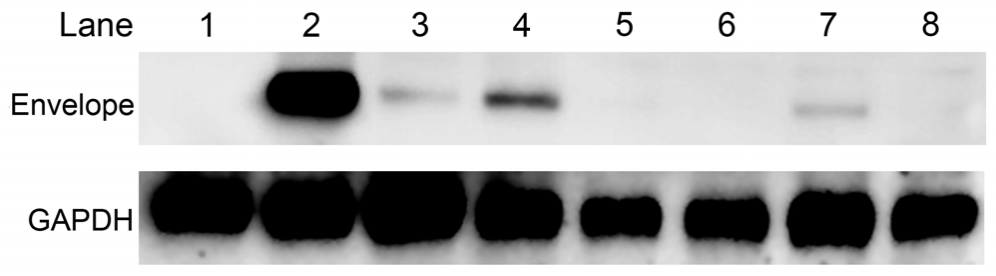
Identification of antiviral effects by western blotting. Expression of JEV E protein in the presence of compounds was examined by western blotting. Protein loading was monitored by the blots of GAPDH. No E protein was detected in the mock-infected cell controls (lane 1). There was an obvious decrease in E protein expression in treated cells (lanes 3–8) compared to the JEV-infected cell controls (lane 2). Lanes 3–5 were cells treated with 10 µM niclosamide, cilnidipine, and FGIN-1-27, respectively. Lanes 6–8 were cells treated with 20 µM niclosamide, cilnidipine, and FGIN-1-27, respectively.

BHK-21 cells infected with JEV were treated with the three compounds at concentrations of 10 and 20 µM. Expression of viral E protein was examined by western blotting. There was no E protein expression in the mock-infected controls (Figure 2, lane 1), whereas an obvious band was found in the JEV-infected cells (Figure 2, lane 2). Expression of E protein was clearly decreased by compounds treatment. No E protein detected in the cilnidipine (20 µM) and FGIN-1-27 (20 and 10 µM) groups (Figure 2, lanes 5, 6, and 8). By gray-value analysis, there were about 85%, 56%, and 75% of E expression decrease by treating with 10 µM niclosamide, 10 µM cilnidipine, and 20 µM cilnidipine respectively (Figure 2, lanes 3, 4, and 7).

#### Identification of antiviral effects by IFA

**Figure 3:**
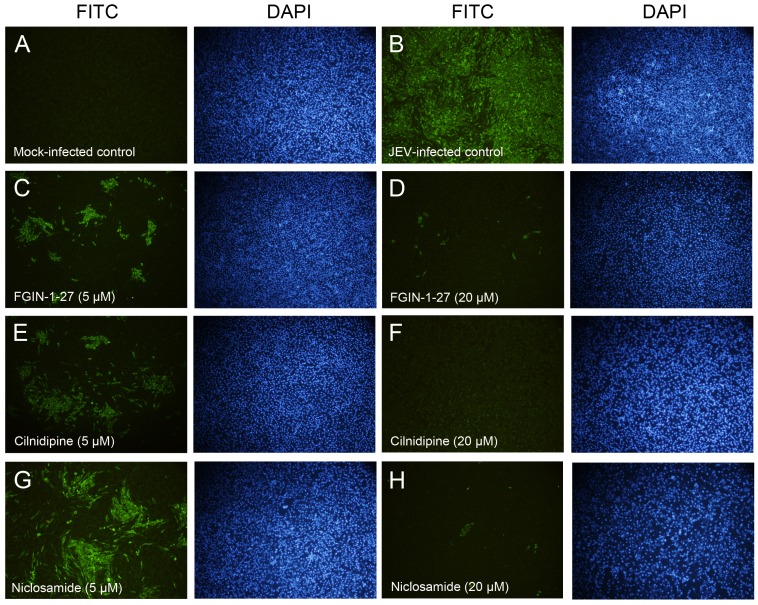
Identification of antiviral effects of the compounds by IFA. The cells were treated with anti-NS5 Mab followed by FITC-conjugated goat anti-mouse antibody, and simultaneously stained with DAPI. The mock-infected cell controls showed NS5-negative (A), while the JEV-infected cells were almost all NS5-positive (B). There were few cells infected with JEV when treated with the compounds at 20 µM (D, F and H). FGIN-1-27 inhibited JEV replication in about 80% cells at a concentration of 5 µM (C). There were ∼60% and ∼30% JEV-negative cells after treatment with 5 µM cilnidipine and niclosamide (E and G).

JEV was propagated in BHK-21 cells in the presence of compounds at 5 or 20 µM. At 48 h post-infection, IFA was performed to assess the reproduction of JEV in the cells. There was no fluorescence signal in the mock-infected cells (Figure 3A), while almost all cells were virus-positive in the JEV-infected cells (Figure 3B). JEV reproduction could be inhibited in the presence of the various compounds at 5 µM concentration. There was about 80%, 60% and 30% JEV-negative cells in the FGIN-1-27-, cilnidipine- and niclosamide-treated groups, respectively (Figure 3C, E and G). Absolute inhibition of JEV replication was achieved when the concentration of cilnidipine reached 20 µM (Figure 3F). Simultaneously, few cells were JEV-positive after treatment with FGIN-1-27 and niclosamide at 20 µM (Figure 3D and H).

#### Identification of antiviral effects by plaque reduction assay

**Figure 4:**
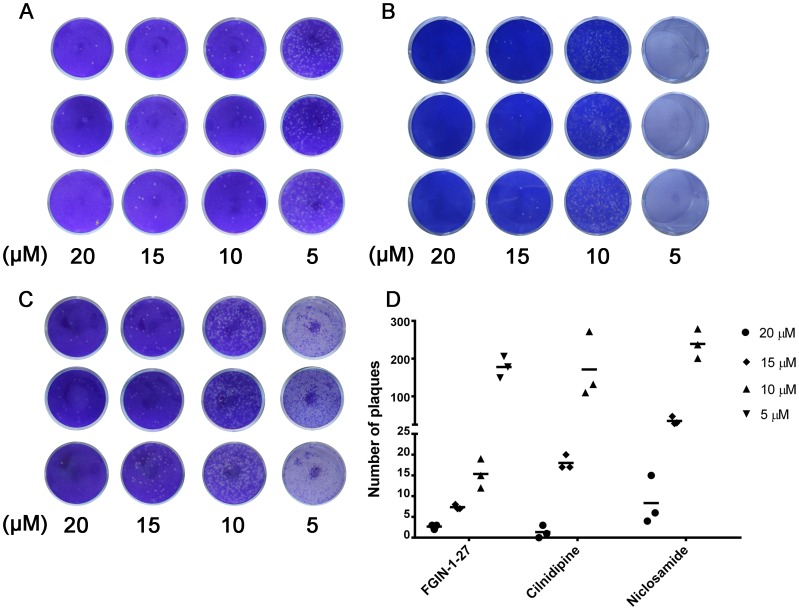
Identification of antiviral effects of the compounds by plaque reduction assay. JEV was propagated in BHK-21 cells in the presence of different concentrations of compounds for 48 h, and the virus titer was tested by plaque assay. A–C show plaque reduction assay of compounds FGIN-1-27, cilnidipine and niclosamide, respectively. The plaque numbers at different concentrations were counted and plotted in panel D.

The JEV-infected cells were treated with different concentrations (20, 15, 10, and 5 µM) of compounds for 48 h. Virus replication was evaluated by plaque assay. The inhibitory effects of the compounds on JEV replication showed a dose-dependent effect (Figure 4A-C). There were no more than 10 plaques (<200 PFU/mL) forming on BHK-21 cells by treating with all the three compounds at 20 µM (Figure 4D). In the FGIN-1-27-treated group, the number of plaques was also <20 (<400 PFU/mL) when the compound concentration was 10 µM (Figure 4A). As a control, the virus titer in the untreated group was 2.7×10^6^ PFU/mL (data not show).

### Time-of-addition assay

**Figure 5:**
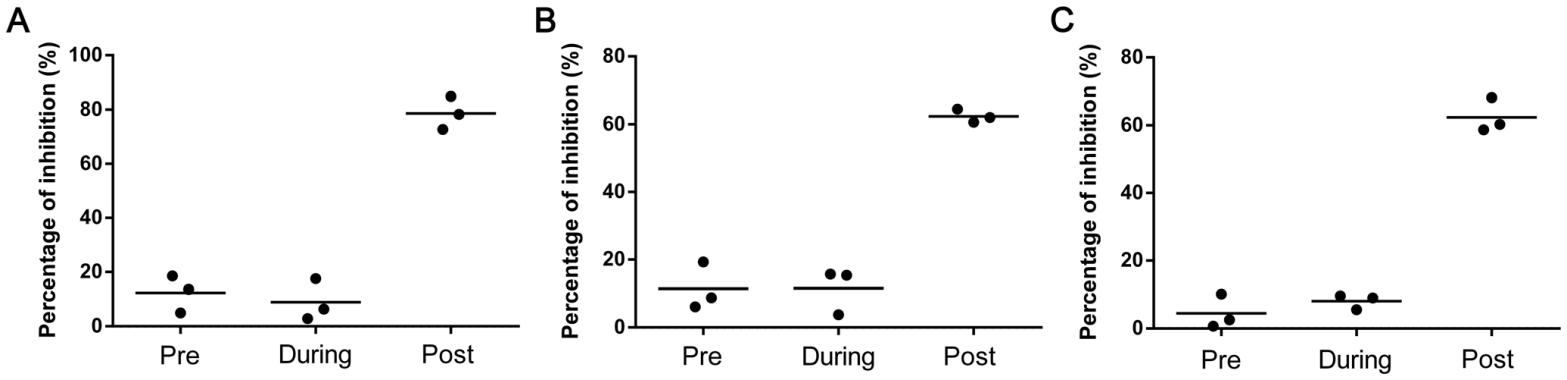
Time-of-addition assay. The antiviral effects of FGIN-1-27 (A), cilnidipine (B) and niclosamide (C) were evaluated pre-, during or post-infection of JEV. All three compounds showed a high inhibition rate at the stage of post-infection.

The compounds were added to JEV-infected cells at −1, 0, and +1 h post-infection, and the percentage inhibition was evaluated after 120 h incubation. No inhibition of infection was detectable when the compounds were added before or during JEV attachment. However, the anti-JEV activities of these compounds were all observed in the post-infection groups (Figure 5A–C). Thus, all three compounds might inhibit JEV at the stage of viral replication.

### EC50 and CC50

**Figure 6:**
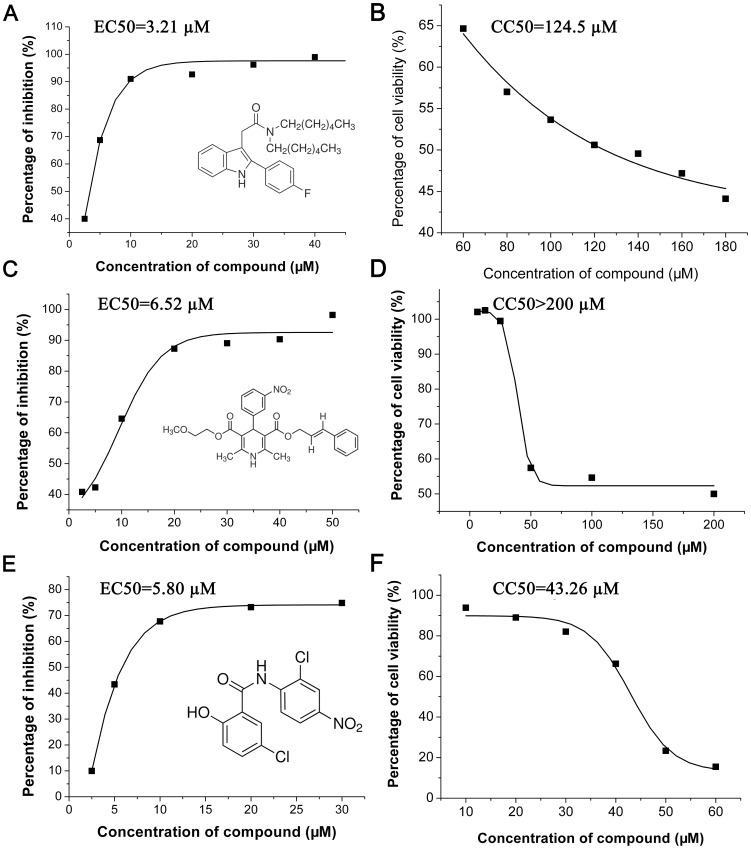
Dose-dependent response of the compounds on JEV replication and cell proliferation. A, C and E show inhibitory effects of different concentrations of FGIN-1-27, cilnidipine and niclosamide, respectively. B, D and F show cell viability in different concentrations of FGIN-1-27, cilnidipine and niclosamide, respectively. EC50s and CC50s were calculated by nonlinear regression. The 2D structures of corresponding compounds are also shown in A, C and E.

To quantify the antiviral effect, the inhibition rates of the three compounds at different concentrations were determined and EC50 was calculated by nonlinear regression. The inhibitory effects of all three compounds showed dose-dependent patterns. The EC50s of FGIN-1-27, cilnidipine and niclosamide were 3.21, 6.52, and 5.80 µM, respectively (Figure 6A, C and E). To assess cytotoxicity, cell viability with different concentrations of compounds was tested and CC50s of FGIN-1-27, cilnidipine and niclosamide were determined to be 124.5, 200, and 43.26 µM (Figure 6B, D and F). So, the selectivity indexes of FGIN-1-27, cilnidipine and niclosamide were 38.79, 30.67 and 7.49, respectively.

## Discussion

To acquire complete CPE induced by JEV, the endpoint of HTS assay was optimized at 120 h post-inoculation. Such a long incubation period posed a real challenge to the robustness and repetitiveness of the HTS assay. After the optimization of cell density and infective dose, this CPE-based HTS showed a high reproducibility including an average *Z*′ value over 0.9, and a suitable S/B ratio around 10.0. Variations from cell control (1.26±0.45%) and virus infected control (5.72±0.23%) were no more than 10%. In addition, the similar *Z*′, S/B, and CV values were obtained in the HTS of 1280 compounds, which demonstrated that this assay was reliable for large scale screening of antiviral inhibitors. Meanwhile, this CPE-based assay could also be used to determine the EC50 of compound on JEV, or quantify the cytopathic effect caused by JEV.

The potential cytotoxicity and inhibition concentration of compounds in a library might be quite different. So in a HTS, to choose one suitable concentration for all compounds is not possible. In the present study, we evaluated the inhibitory effects of each compound in three concentrations (50, 25 and 12.5 µM), and some compounds were found to have higher inhibition rate at 25 µM than 50 µM. The compound in high concentration might influence the cell viability and thus exhibited a lower inhibition rate. Especially in the CC50 analysis, the three tested compounds were proven to have more or less cytotoxicity at 50 µM to cells (Fig. 6B, D and F). So it is not difficult to understand why the inhibition rate of some compounds at 50 µM is lower than that at 25 µM.

The antiviral mechanism of the three hit compounds was explored in this study. All three compounds showed inhibitory effects at the post-infection stage, using the time-of-addition assay (Figure 5). Therefore, these compounds should inhibit virus in the process of replication. The essential viral proteins related to replication include NS3 protease, NS3 helicase, methyltransferase (MTase) and RNA-dependent RNA polymerase (RdRp) [25]. In our study, the inhibitory effects of the compounds on JEV NS3 protease and helicase were tested by fluorescence resonance energy transfer (FRET) respectively, as previously described [26], [27], but the compounds did not inhibit these two proteins (unpublished data). Then the potential antiviral targets may be the MTase, RdRp or other cellular enzymes. How the compounds inhibit JEV replication was under further investigation.

Cilnidipine is a dual blocker of L- and N-type calcium channels in vascular smooth muscle or sympathetic nerve terminals that supply blood vessels [28]. It is effective for treatment of essential hypertension and has been approved in Japan [29]. The toxicity of cilnidipine is low (MLD50 >5 g/kg) and has a low incidence of unfavorable side effects in humans [30]. In the present study, cilnidipine also showed effective inhibition of JEV. The replication of JEV was almost completely inhibited by 20 or 15 µM cilnidipine. So, cilnidipine might be a candidate anti-JEV drug.

FGIN-1-27 is an anxiolytic drug acting on the peripheral benzodiazepine receptor, producing anxiolytic effects by stimulating steroidogenesis of neuroactive steroids such as allopregnanolone [31]. In the present study, FGIN-1-27 showed ideal antiviral effects at concentrations of 5–20 µM. The high selectivity index (38.78) illustrated that FGIN-1-27 could inhibit JEV with high specificity. A previous study showed that FGIN-1-27 had the ability to enter the brain [32]. Therefore, FGIN-1-27 might inhibit JEV in brain cells, and could be a potential drug for treatment of encephalitis caused by JEV.
